# Supplementary material for: Reaching the precision limit with tensor-based wavefront shaping
Source: Nat Commun. 2024 Jul 26;15:6319. doi: 10.1038/s41467-024-50513-8 (PMC11282273; doi:10.1038/s41467-024-50513-8)
Supplement: Supplementary file 1 — Supplementary Information [file 41467_2024_50513_MOESM1_ESM.pdf]

Supplementary information for *Reaching the precision limit with tensor-based wavefront shaping*

Rodrigo Gutiérrez-Cuevas,[1]* Dorian Bouchet,[2] Julien de Rosny,[1] and Sébastien M. Popoff[1]

[1]Institut Langevin, ESPCI Paris, Université PSL, CNRS, 75005 Paris, France
[2]Université Grenoble Alpes, CNRS, LIPhy, Grenoble, France
*rodrigo.gutierrez-cuevas@espci.fr

28 June 2024

Section 1 contains the derivations for all the expressions for the Fisher information presented in the main text. Section 2 provides a detailed summary of the tools necessary to find the best rank-one approximation of higher-order tensors such as the higher-order singular value decomposition and the alternating least squares algorithm. Section 3 shows how to optimize the Fisher information when the output projection modes can be changed. Section 4 gives details about the experimental implementation for measuring the transmission matrix of the fiber and the related tensors. Section 5 provides details about the characterization of noise in the setup and the estimation of small deformations. Section 6 studies how the dependence of the Fisher information for various channels changes when the deformation is performed at another location.

1 Derivations for the Fisher information expressions

1.1 Fisher information for intensity measurements

Given a random process determined by a probability mass function $P(\chi|\zeta)$ for the set of random variables χ and with parametric dependence on ζ, the Fisher information for the parameter ζ is defined as

$$\mathcal{J}(\zeta) = \overline{\left[\partial_\zeta \ln P(\chi|\zeta)\right]^2}, \tag{S1}$$

where the overline denotes the expected values over all the possible values of χ [1,2]. Here, $P(\chi|\zeta)$ represents the inaccuracy of the measured intensity distribution over the N output modes $\{\mathbf{y}_1,\dots,\mathbf{y}_N\}$ due to the presence of noise. Thus, the measured intensity distribution is given by $\chi = \mathbf{I} + \boldsymbol{w}$ where $\mathbf{I} = |\mathbf{e}^{(\mathrm{out})}|^2$ is the expected intensity distribution and $\boldsymbol{w}$ represents the noise which is assumed to be Gaussian with known standard deviation σ_i for the ith output mode. The probability mass function can then be written as

$$P(\chi|\zeta) = \prod_{i=1}^{N} \frac{1}{\sigma_i\sqrt{2\pi}} e^{-\frac{(\chi_i - I_i)^2}{2\sigma_i^2}}, \tag{S2}$$

The intensity value of the nth output mode is defined as $I_n = |e_n^{(\mathrm{out})}|^2$ where $e_n^{(\mathrm{out})}$ is the coefficient of the nth output mode in the linear expansion $\mathbf{e}^{(\mathrm{out})} = \sum_n e_n^{(\mathrm{out})}\mathbf{y}_n$. The Fisher information then takes the following simple form

$$\mathcal{J}(\zeta) = \sum_{i=1}^{N} \frac{1}{\sigma_i^2}\left[\partial_\zeta I_i\right]^2. \tag{S3}$$

1.2 Fisher information as a rank-one approximation

Writing the output field in terms of the matrix $\mathbf{H}$ and the input field, which is decomposed in terms of the input modes $\{\mathbf{x}_1, \ldots, \mathbf{x}_M\}$, $\mathbf{e}^{(\mathrm{in})} = \sum_{j=1} e_j^{(\mathrm{in})}\mathbf{x}_j$, and normalized, $||\mathbf{e}^{(\mathrm{in})}|| = 1$, we get that

$$\mathcal{J}(\zeta) = \frac{1}{\sigma^2}\sum_{i=1}^{N}\left[\sum_{j,k=1}^{M} \partial_\zeta (\mathrm{H}_{ij}\mathrm{H}_{ik}^*) e_j^{(\mathrm{in})} e_k^{(\mathrm{in})*}\right]^2 \tag{S4a}$$

$$= \frac{1}{\sigma^2}\sum_{i=1}^{N}\left[\sum_{j,k=1}^{M} \mathcal{W}_{ijk}^{(3)*} e_j^{(\mathrm{in})} e_k^{(\mathrm{in})*}\right]^2, \tag{S4b}$$

where $\mathcal{W}_{ijk}^{(3)} = \mathrm{H}_{ij}^*\mathrm{H}_{ik}$ is a third-order tensor encoding the nonlinear relationship between the input field and the changes in intensity induces by first-order variations of the parameter ζ. For simplicity, we have taken the noise over the output modes to be the same, $\sigma = \sigma_i$ for all i, but, if needed, these output mode-dependent noise variations can be incorporated in the definition of $\boldsymbol{\mathcal{W}}^{(3)}$. It is possible to keep rewriting this expression for the Fisher information to get a more insightful expression,

$$\mathcal{J}(\zeta) = \frac{1}{\sigma^2}\sum_{i=1}^{N}\left[\sum_{j',k'=1}^{M} \mathcal{W}_{ij'k'}^{(3)*} e_{j'}^{(\mathrm{in})} e_{k'}^{(\mathrm{in})*}\right]\left[\sum_{j,k=1}^{M} \mathcal{W}_{ijk}^{(3)*} e_j^{(\mathrm{in})} e_k^{(\mathrm{in})*}\right], \tag{S5a}$$

$$= \frac{1}{\sigma^2}\sum_{i=1}^{N}\sum_{j,k=1}^{M} \mathcal{W}_{ijk}^{(3)*} a_i e_j^{(\mathrm{in})} e_k^{(\mathrm{in})*}, \tag{S5b}$$

where we introduced the real vector $\mathbf{a}$ defined component-wise as

$$a_i = \frac{1}{\sigma}\sum_{j,k=1}^{M} \mathcal{W}_{ijk}^{(3)*} e_j^{(\mathrm{in})} e_k^{(\mathrm{in})*}. \tag{S6}$$

This real vector $\mathbf{a}$ is proportional to the rate of change of the intensity distribution over the output modes with respect to the parameter ζ for the input field $\mathbf{e}^{(\mathrm{in})}$. Its value is fixed as the one that maximizes $\mathcal{J}$ for a fixed $\mathbf{e}^{(\mathrm{in})}$. It is easy to show that the $\|\mathbf{a}\| = \mathcal{J}^{1/2}(\zeta)$ so that substituting $\mathbf{a}$ by $\tilde{\mathbf{a}} = \mathbf{a}/\|\mathbf{a}\|$ in the expression for the Fisher information we get,

$$\mathcal{J}(\zeta) = \frac{\mathcal{J}^{1/2}(\zeta)}{\sigma}\sum_{i=1}^{N}\sum_{j,k=1}^{M} \mathcal{W}_{ijk}^{(3)*} \tilde{a}_i e_j^{(\mathrm{in})} e_k^{(\mathrm{in})*} \tag{S7a}$$

$$\frac{\mathcal{J}^{1/2}(\zeta)}{\sigma}\left\langle \boldsymbol{\mathcal{W}}^{(3)}, \tilde{\mathbf{a}} \otimes \mathbf{e}^{(\mathrm{in})} \otimes \mathbf{e}^{(\mathrm{in})*}\right\rangle, \tag{S7b}$$

Here, $\langle \ldots, \ldots \rangle$ denotes the inner product of two tensors as defined in Eq. (S19). Finally, by squaring both sides of Eq. S7b, the Fisher information can be rewritten as

$$\mathcal{J}(\zeta) = \frac{1}{\sigma^2}\left\langle \boldsymbol{\mathcal{W}}^{(3)}, \tilde{\mathbf{a}} \otimes \mathbf{e}^{(\mathrm{in})} \otimes \mathbf{e}^{(\mathrm{in})*}\right\rangle^2. \tag{S8}$$

Given that $||\tilde{\mathbf{a}}|| = ||\mathbf{e}^{(\mathrm{in})}|| = 1$, this expression can almost be seen as a rank-one approximation of the third-order tensor $\boldsymbol{\mathcal{W}}^{(3)}$ [3]. The difference lies on the fact that $\tilde{\mathbf{a}}$ depends on the input field $\mathbf{e}^{(\mathrm{in})}$ and the tensor we are trying to approximate $\boldsymbol{\mathcal{W}}^{(3)}$.

Let us instead consider the cost function

$$C = \left\langle \mathcal{W}^{(3)}, \mathbf{u} \otimes \mathbf{v} \otimes \mathbf{v}^* \right\rangle^2, \tag{S9}$$

where $||\mathbf{u}|| = ||\mathbf{v}|| = 1$, and $\mathbf{u}$ is independent of $\mathbf{v}$. This cost function has more degrees of freedom than $\mathcal{J}$, and is easy to show that $C = \sigma^2 \mathcal{J}$ if

$$u_n = \frac{\sum_{mm'} \mathcal{W}^{(3)}_{nmm'}{}^* v_m v^*_{m'}}{\sum_n \left(\sum_{mm'} \mathcal{W}^{(3)}_{nmm'}{}^* v_m v^*_{m'} \right)^2}, \tag{S10}$$

for $\mathbf{e}^{(\text{in})} = \mathbf{v}$. Now, if we find $\mathbf{u}$ and $\mathbf{v}$ such that C is a maximum then it is easy to verify that $\mathbf{u}$ is related to $\mathbf{v}$ according to Eq. (S10) and

$$\max_{\mathbf{e}^{(\text{in})}} \mathcal{J} = \frac{1}{\sigma^2} \max_{\mathbf{u},\mathbf{v}} C. \tag{S11}$$

Moreover, since $\mathcal{J}$ lives in a smaller subspace than C, at these points $\mathcal{J}$ is also at a maxima for $\mathbf{e}^{(\text{in})} = \mathbf{v}$. This demonstrates that the input field that maximizes the Fisher information can be found by computing the best rank-one approximation of $\mathcal{W}^{(3)}$.

1.3 Changing the output projection modes

To project the output field onto a set of Q orthonormal output projection modes (OPM), we simply apply the corresponding projection operator $\mathbf{P}$ to the output field. The projection operator satisfies $\mathbf{P}^\dagger \cdot \mathbf{P} = \mathbb{1}_Q$ where $\mathbb{1}_Q$ is the identity matrix of dimension Q. Note that $\mathbf{P}$ is generally not unitary since Q can be less than the total number of output modes in the system. The projected output field can then be written as

$$\mathbf{e}_P^{(\text{out})} = \mathbf{P}^\dagger \cdot \mathbf{e}^{(\text{out})} = \sum_{q=1}^{Q} e_{P,q}^{(\text{out})} \mathbf{p}_q, \tag{S12}$$

where $\mathbf{p}_q$ is the qth column of $\mathbf{P}$, i.e. the qth OPM, and the intensity distribution over the new OPM is given by $I_q^{(P)} = |e_{P,q}^{(\text{out})}|^2$. Rewriting the Fisher information in terms of an output modes basis that contains all the information about the output field we have that,

$$\mathcal{J}(\zeta) = \frac{1}{\sigma^2} \sum_{q=1}^{Q} \left[\partial_\zeta |e_{P,q}^{(\text{out})}|^2 \right]^2, \tag{S13a}$$

$$= \frac{1}{\sigma^2} \sum_{q=1}^{Q} \left[\sum_{n,n'} P^*_{nq} \partial_\zeta (e_n^{(\text{out})} e_{n'}^{(\text{out})*}) P_{n'q} \right]^2, \tag{S13b}$$

$$= \frac{1}{\sigma^2} \sum_{q=1}^{Q} \langle \mathbf{p}_q, \mathbf{E}_\zeta \cdot \mathbf{p}_q \rangle^2, \tag{S13c}$$

where

$$\mathbf{E}_\zeta = \partial_\zeta \left(\mathbf{e}^{(\text{out})} \otimes \mathbf{e}^{(\text{out})*} \right). \tag{S14}$$

It is easy to see that the $\mathbf{E}_\zeta$ operator is Hermitian and of rank two. Therefore, it only has two non-zero eigenvalues, which means that all the information available in $\mathbf{e}^{(\text{out})}$ can be obtained by projecting into two modes. Note that this result applies even to changes happening at the source and not only those encoded in the matrix $\mathbf{H}$.

1.4 Fisher information for optimal input-output combinations

Now, if we want to find the optimal input-output combination in order to reach the precision limit for intensity-based measurements, then we need to write the Fisher information in terms of the input field. Moreover, as we just showed, we only need to consider projection the output into two OPM. Therefore, setting $\mathbf{e}^{(\mathrm{out})} = \mathbf{H} \cdot \mathbf{e}^{(\mathrm{in})}$ and $Q = 2$ in Eq. (S13b), we get

$$\mathcal{J}(\zeta) = \frac{1}{\sigma^2} \sum_{q=1}^{Q} \left[\sum_{n,n'} \sum_{mm'} \partial_\zeta (\mathrm{H}_{nm} \mathrm{H}^*_{n'm'}) P^*_{nq} P_{n'q} e^{(\mathrm{in})}_m e^{(\mathrm{in})*}_{m'} \right]^2, \tag{S15a}$$

$$= \frac{1}{\sigma^2} \sum_{q=1}^{Q} \langle \mathcal{W}^{(4)}, \mathbf{p}^*_q \otimes \mathbf{p}_q \otimes \mathbf{e}^{(\mathrm{in})} \otimes \mathbf{e}^{(\mathrm{in})*} \rangle^2, \tag{S15b}$$

where we defined the fourth-order tensor $\mathcal{W}^{(4)}$ whose components are given by

$$\mathcal{W}^{(4)}_{ijkl} = \partial_\zeta (\mathrm{H}^*_{ik} \mathrm{H}_{jl}). \tag{S16}$$

Another way to understand the dependence of the Fisher information on the OPMs is by noticing that the tensor $\mathcal{W}^{(3)}$ does not follow the standard transformation rules when the output basis is changes. This is in part due to the fact that the output space corresponds to changes in intensity which is a different physical quantity than the output field. For this reason, when including the OPMs in the optimization it becomes necessary to introduce the fourth-order tensor $\mathcal{W}^{(4)}$ which does follow the standard transformation rules under changes of the output basis.

2 Finding the best rank-one approximation

2.1 Best rank-one tensor approximation

Let us consider a tensor $\mathcal{T} \in \mathbb{C}^{I_1} \times \cdots \times \mathbb{C}^{I_L}$ of order L. The best rank-one approximation of $\mathcal{T}$ is defined as the rank-one tensor that solves the following minimization problem

$$\min_{\mathbf{u}^{(1)},\cdots,\mathbf{u}^{(L)}} \left\| \mathcal{T} - \mathbf{u}^{(1)} \otimes \cdots \otimes \mathbf{u}^{(L)} \right\|^2 = \min_{\mathbf{u}^{(1)},\cdots,\mathbf{u}^{(L)}} \sum_{i_1,\cdots,i_L} \left(\mathcal{T}_{i_1,\cdots,i_L} - u^{(1)}_{i_1} \cdots u^{(L)}_{i_L} \right)^2. \tag{S17}$$

It is possible to show that this minimization problem is equivalent to the following maximization problem (see Ref. [3] for proof),

$$\max_{\mathbf{u}^{(1)},\cdots,\mathbf{u}^{(L)}} \left| \langle \mathcal{T}, \mathbf{u}^{(1)} \otimes \cdots \otimes \mathbf{u}^{(L)} \rangle \right|^2 \text{ subject to } \left\| \mathbf{u}^{(i)} \right\| = 1 \quad \forall \quad i \in \{1, \ldots, L\}, \tag{S18}$$

where $\langle \cdots, \cdots \rangle$ denotes the inner product induced by the Frobenius norm, namely

$$\langle \mathcal{T}^{(1)}, \mathcal{T}^{(2)} \rangle = \sum_{i_1=1}^{I_1} \cdots \sum_{i_L=1}^{I_L} \mathcal{T}^{(1)*}_{i_1 \cdots i_L} \mathcal{T}^{(2)}_{i_1 \cdots i_L}. \tag{S19}$$

Finding the best rank-one approximation is a nonlinear problem for which finding the optimal solution is not as straightforward as it is for matrices.

Algorithm 1 | Higher-order singular value decomposition

Input

$\mathcal{T}$	tensor of order L

Output

$[\mathbf{U}^{(1)}, \dots, \mathbf{U}^{(L)}]$	list of matrices containing the higher-order singular vectors
$\mathcal{S}$	core tensor

Procedure

```
S ← T                                  ▹ initialize the core tensor
for n ← 1 to L do
    T^(n) ← unfold(T, n)               ▹ compute the n-mode unfolding of T
    U, s, V ← svd(T^(n))               ▹ computes singular value decomposition
    U^(n) ← U                          ▹ assign the left singular vectors as the higher-order singular vectors
    S ← T ×_n U^(n)†                   ▹ update core tensor by computing the n-mode product
end for
```

2.2 Higher-order singular value decomposition.

A first step into the right direction for finding the best rank-one approximation can be taken by computing the HOSVD which is defined in what follows. A tensor $\mathcal{T} \in \mathbb{C}^{I_1} \times \cdots \times \mathbb{C}^{I_L}$ of order L can be decomposed as a sum of rank-one tensors using a higher-order version of the SVD,

$$\mathcal{T} = \sum_{i_1=1}^{I_1} \cdots \sum_{i_L=1}^{I_L} \mathcal{S}_{i_1 \cdots i_L} \mathbf{u}_{i_1}^{(1)} \otimes \cdots \otimes \mathbf{u}_{i_L}^{(L)} \tag{S20}$$

where the higher-order singular vectors $\mathbf{u}_i^{(j)}$ form an orthonormal basis of $\mathbb{C}^{I_j}$, $\mathcal{S}_{i_1 \cdots i_L}$ are the entries of the core tensor $\mathcal{S}$, and $\otimes$ denotes the outer product. The core tensor satisfies the properties of

1. all orthogonality: $\langle \mathcal{S}_{i_n=\alpha}, \mathcal{S}_{i_n=\beta} \rangle = 0$ for $\alpha \neq \beta$
2. ordering: $\left\| \mathcal{S}_{i_n=1} \right\| \geq \left\| \mathcal{S}_{i_n=2} \right\| \geq \dots,$

where $\mathcal{S}_{i_n=\alpha}$ denotes the tensor obtained by setting the nth index equal to α. For matrices, these properties reduce to the diagonality condition of the matrix of singular values and their ordering.

Another important property of the HOSVD is that it preserves the partial symmetries of the original tensor, for example if $\mathcal{T}_{i_1 \cdots i_j \cdots i_k \cdots i_L} = \mathcal{T}^*_{i_1 \cdots i_k \cdots i_j \cdots i_L}$ then $\mathbf{u}_i^{(j)} = \mathbf{u}_i^{(k)*}$ and $\mathcal{S}_{i_1 \cdots i_j \cdots i_k \cdots i_L} = \mathcal{S}^*_{i_1 \cdots i_k \cdots i_j \cdots i_L}$. The biggest advantage of the HOSVD against other types of tensor decompositions is that it can be easily computed via the SVD of its matrix unfoldings (see Algorithm 1 and Refs. 4, 5 for more details). However, when truncating the HOSVD to a single term we do not get the best rank one approximation, but we do get an excellent first guess.

2.3 Alternating least-squares algorithm

Finding the best rank-one approximation is a particular case of more general types of lower-rank tensor approximations based around the polyadic and Tucker decompositions [6, 7]. There are several algorithms

Algorithm 2 | Alternating least squares

Input

$\mathcal{T}$	tensor of order L
$[\mathbf{u}^{(1)}, \ldots, \mathbf{u}^{(L)}]$	initial guess for the best-rank-one approximation
$\mathcal{P}$	symmetries of the tensor
N_{iter}	number of iterations

Output

$[\mathbf{u}^{(1)}, \ldots, \mathbf{u}^{(L)}]$	list of vectors providing the best-rank-one approximation

Procedure

```
1: L ← order(T)                                              ▷ get the order of the tensor
2: for n ← 1 to N_iter do
3:     for m ← 1 to L do
4:         u^(m) ← min_{u^(m)} ||T − u^(1) ⊗ ··· ⊗ u^(L)||^2    ▷ solve least squares minimization
5:     end for
6:     [u^(1), ..., u^(L)] ← symmetrize([u^(1), ..., u^(L)], P)   ▷ impose symmetries
7: end for
```

that have been developed to find them and that could, in principle, be applied to solve the optimization problem in Eq. (S17). Here, we use the iterative alternating-least squares (ALS) algorithm and use as a starting point the first higher-order singular vectors. For the ALS algorithm, instead of tackling the minimization over **a**, **b**, and **c** all at once, we iterate over each vector by solving the standard least-square problem that results from leaving the other ones fixed (see Algorithm 2). For example, we start by solving

$$\min_{\mathbf{a}} \|\mathcal{T} - \mathbf{a} \otimes \mathbf{b} \otimes \mathbf{c}\|, \tag{S21}$$

for which we can find the exact solution. Then, we do the same for **b** and **c**. This is repeated for a predefined number of times until convergence is reached.

One issue that can arise from the solution is that it does not satisfy the symmetries of the original tensor. Here, we were able to adapt the ALS algorithm in order to keep the symmetries of the original tensor if needed. Let us consider the case of the third-order tensor $\mathcal{W}^{(3)}$ which has a partial Hermitian symmetry in the last two indices, $\mathcal{W}^{(3)}_{ijk} = \mathcal{W}^{(3)*}_{ikj}$, where we seek the vectors **a** and **b** that solve

$$\min_{\mathbf{a},\mathbf{b}} \|\mathcal{T} - \mathbf{a} \otimes \mathbf{b} \otimes \mathbf{b}^*\|. \tag{S22}$$

Initially, we do not force the symmetry and allow the last vector to be different, say **c** and start by minimizing $\|\mathcal{T} - \mathbf{a} \otimes \mathbf{b} \otimes \mathbf{c}\|$. We proceed as a standard ALS routine by fixing **b** and **c**, and solving the minimization problem

$$\min_{\mathbf{a}} \|\mathcal{T} - \mathbf{a} \otimes \mathbf{b} \otimes \mathbf{c}\|, \tag{S23}$$

which is a standard least-squares problem with a fixed solution. Then, we do the same for **b** and **c**. Before

performing another loop, we symmetrize the lower rank tensor by setting

$$\mathbf{b} \leftarrow \frac{\sqrt{\|\mathbf{b}\|\|\mathbf{c}\|}}{2}\left(\frac{\mathbf{b}}{\|\mathbf{b}\|}+\frac{\mathbf{c}^*}{\|\mathbf{c}\|}\right) \tag{S24a}$$

$$\mathbf{c} \leftarrow \mathbf{b}^* \tag{S24b}$$

This simple adaptation gives enough freedom so the algorithm converges and provides a solution with the desired symmetry. A similar approach is taken to compute the rank-one approximation of $\mathcal{W}^{(4)}$.

3 Including the output projection modes in the Fisher information optimization

3.1 Finding the optimal output projection modes

As demonstrated in Sec. 1.3, for a fixed input field the optimal OPMs are given in terms of the Hermitian matrix,

$$\mathbf{E}_\zeta = \partial_\zeta\left(\mathbf{e}^{(\mathrm{out})} \otimes \mathbf{e}^{(\mathrm{out})*}\right) = \alpha\left(\mathbf{e}_1 \otimes \mathbf{e}_2^* + \mathbf{e}_2 \otimes \mathbf{e}_1^*\right), \tag{S25}$$

where $\mathbf{e}_1 = \mathbf{e}^{(\mathrm{out})}/\left\|\mathbf{e}^{(\mathrm{out})}\right\|$ and $\mathbf{e}_2 = \partial_\zeta \mathbf{e}^{(\mathrm{out})}/\left\|\partial_\zeta \mathbf{e}^{(\mathrm{out})}\right\|$, and $\alpha = \left\|\mathbf{e}^{(\mathrm{out})}\right\|\left\|\partial_\zeta \mathbf{e}^{(\mathrm{out})}\right\|$. In order to find these optimal OPMs, we first need to write this matrix in terms of an orthogonal basis. Moreover, since due to its rank only two elements suffice. Using the normalized output field $\mathbf{e}_1$ and the normalized orthogonal component of $\mathbf{e}_2$ we have that,

$$\mathbf{E}_\zeta = \alpha\left[(\rho+\rho^*)\mathbf{e}_1 \otimes \mathbf{e}_1^* + \sqrt{1-|\rho|^2}\left(\mathbf{u} \otimes \mathbf{e}_1^* + \mathbf{e}_1 \otimes \mathbf{u}^*\right)\right], \tag{S26}$$

where

$$\mathbf{u} = \frac{1}{\sqrt{1-|\rho|^2}}(-\rho\mathbf{e}_1 + \mathbf{e}_2) \qquad \text{and} \qquad \mathbf{e}_1^* \cdot \mathbf{u} = 0 \qquad \text{and} \qquad \mathbf{e}_1^* \cdot \mathbf{e}_2 = \rho = \rho_r + \mathrm{i}\rho_i, \tag{S27}$$

with ρ_r and ρ_i being the real and imaginary parts of ρ, respectively. The two non-zero eigenvalues can then be found to be given by

$$\lambda_\pm = \alpha\left(\rho_r \pm \sqrt{1-|\rho|^2+\rho_r^2}\right), \tag{S28}$$

where it is easy to see that $\lambda_+ \geq 0$, and the corresponding normalized eigenvectors are

$$\mathbf{v}_\pm = \beta_\pm\left[\left(\rho_r \pm \sqrt{1-|\rho|^2+\rho_r^2}\right)\mathbf{e}_1 + \sqrt{1-|\rho|^2}\mathbf{u}\right], \tag{S29}$$

with

$$\beta_\pm = 1/\sqrt{2\left[1-|\rho|^2+\rho_r(\rho_r \pm \sqrt{1-|\rho|^2+\rho_r^2})\right]}. \tag{S30}$$

Now, the Fisher information in Eq. (S13c) can be rewritten as

$$\mathcal{J}(\zeta) = \frac{1}{\sigma^2}\sum_{q=1}^{2}\langle \mathbf{p}_q, \left(\lambda_+\mathbf{v}_+ \otimes \mathbf{v}_+^* + \lambda_-\mathbf{v}_- \otimes \mathbf{v}_-^*\right) \cdot \mathbf{p}_q\rangle^2. \tag{S31}$$

The optimal OPMs need to be given by a linear combination of the eigenvectors and be orthogonal between them. Therefore, we can write them as,

$$\mathbf{p}_1 = \cos\theta \mathbf{v}_+ + \sin\theta e^{\mathrm{i}\phi}\mathbf{v}_-, \qquad \mathbf{p}_2 = \sin\theta e^{-\mathrm{i}\phi}\mathbf{v}_+ - \cos\theta \mathbf{v}_-. \tag{S32}$$

Substituting these expressions into the Fisher information we get,

$$\mathcal{J}(\zeta) = \frac{1}{\sigma^2}\left[\left(\cos^2\theta\lambda_+ + \sin^2\theta\lambda_-\right)^2 + \left(\sin^2\theta\lambda_+ + \cos^2\theta\lambda_-\right)^2\right], \tag{S33}$$

which is a periodic function with period $\pi/2$. Taking the first and second derivatives with respect to θ, we get

$$\partial_\theta \mathcal{J}(\zeta) = -(\lambda_+ - \lambda_-)^2 \sin(4\theta), \tag{S34}$$

$$\partial_\theta^2 \mathcal{J}(\zeta) = -4(\lambda_+ - \lambda_-)^2 \cos(4\theta). \tag{S35}$$

Given that $\lambda_+ - \lambda_- = 2\alpha\sqrt{1 - |\rho|^2 + \rho_r^2} > 0$, the Fisher information attains it maxima for $\theta = 0$, thus showing that the optimal OPMs are the eigenvectors of $\mathbf{E}_\zeta$,

$$\mathbf{p}_1^{(\text{optimal})} = \mathbf{v}_+, \qquad \mathbf{p}_2^{(\text{optimal})} = \mathbf{v}_-. \tag{S36}$$

For small perturbations and unitary systems in which the perturbation does not induce losses, it can be shown that $\rho_r = 0$. In this case, the eigenvectors are given by the symmetric and antisymmetric combinations of the output field $\mathbf{e}^{(\text{out})}$ and the orthogonal component of the derivative $\partial_\zeta \mathbf{e}^{(\text{out})}$.

3.2 Finding the optimal input-output combination

The first step in finding the optimal input-output combination is to compute the fourth-order tensor $\mathcal{W}^{(4)}$. Given the number of pixels used as output modes to measure $\mathbf{H}$, the size of this tensor would be too large to be useful for computations. However, since the number of input modes is much lower, one can see that using all the 1444 output pixels might be an overkill. Therefore, we first compute the SVD for the two TMs that we used to compute $\mathcal{W}^{(3)}$ and use their output singular vectors to form an orthonormal set of output modes. This new set of output modes has double the number of input modes used, but it allows us to capture all the information encoded in the pixel basis but at a much smaller size. Then, we project the TMs onto this set of output modes and use this projected TMs to compute $\mathcal{W}^{(4)}$.

As mentioned in the main text, when trying to find the optimal input-output combination that allows reaching the precision limit, the Fisher information given by Eq. (6) of the main text no longer takes the form of a lower-rank tensor decomposition. Nonetheless, it is possible to rewrite it as a nonlinear optimization where only the expansion coefficients of the input field in terms of the input modes are used as optimization parameters. This is done by first computing the output field using the TM, then getting the optimal OPMs and using those to compute the Fisher information. This optimization was implemented using the neural network framework PyTorch. Here again, we need to provide a first guess, and, as for the ALS algorithm, we chose to use the first term of the HOSVD of $\mathcal{W}^{(4)}$.

4 Experimental characterization of an MMF under perturbation

4.1 Experimental setup

The optical setup is represented in Fig. S1. The light source consists of a continuous linearly polarized laser beam at 1550 nm (TeraXion NLL) injected into a 10:90 polarization-maintaining fiber coupler (PNH1550R2F1) in order to separate it into the shaped signal field and the reference field. The 90% arm

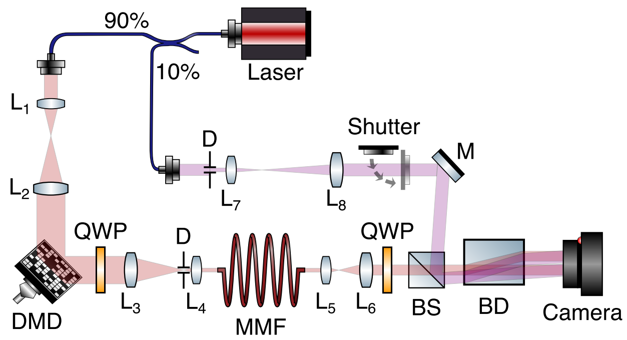

Fig. S1 | Optical setup. DMD, digital micromirror device; L_i, lenses; QWP, quarter-wave plates; M, mirror; D, diaphragm; BD, beam displacer; BS, beam splitter. The shutter is used to block the reference beam in order to perform intensity measurements.

is collimated and expanded to illuminate a digital micromirror device (DMD) (Vialux V-650L) which is used to modulate the signal field in amplitude and phase using Lee holograms [8, 9]. The light is converted into left circular polarization using a quarter-wave plate. The shaped signal field is then imaged with a 4f system onto the input facet of a 25 cm-long step-index fiber with a 50 μm core and 0.22 numerical aperture, which is held approximately straight. The output facet is imaged via another 4f system onto an InGaAs camera (Xenics Cheetah 640-CL 400 Hz) after passing through a quarter-wave plate, followed by a beam displacer to select the left-circularly polarized component. The other 10% arm is used to produce a tilted reference that is made to interfere with the signal field in order to be able to retrieve the output field via off-axis holography [10]. An Arduino-controlled shutter allows blocking the reference field to perform intensity measurements of the signal field. The fiber can be deformed, roughly in the middle, by pressing on it using a 50 nm precision dc servo motor actuator (Thorlabs Z812). All the values reported for the deformation correspond to the linear displacement of the actuator and not the deformation of the fiber core, which is much smaller since most of the deformation is absorbed by the coating of the fiber.

4.2 TM and $\mathcal{W}^{(3)}$ measurements

First, we measure the TM of the MMF in the pixel basis, by sending 7200 square layouts consisting of 37×37 square macropixels whose value is either zero or a random phase of amplitude one. Each macropixel is formed by grouping 16×16 pixels of the DMD, where the desired phase and amplitude value is encoded via Lee holograms [8] and selecting the first order of diffraction at the Fourier plane. The corresponding output fields are recovered from the interferograms between the reference and signal fields, and subsequently projected onto a square pattern of 44×44 macropixels formed by grouping 4×4 pixels of the camera. Regrouping all input and output fields into the columns of matrices $\mathbf{X}$ and $\mathbf{Y}$, respectively, we reconstruct the TM via $\mathbf{H} = \mathbf{Y} \cdot \mathbf{X}^{-1}$ where $\mathbf{X}^{-1}$ denotes the pseudoinverse of $\mathbf{X}$.

Then, we compute the SVD of the TM in the pixel basis and use it to identify the fiber modes as the singular vectors with singular values in the almost constant plateau (see Fig. S2). For the fiber we used there were 144 fiber modes as opposed to the 129 predicted through numerical simulations. This indicates that some cladding modes are sufficiently close to the cutoff that they propagate without undergoing significant absorption for the short length we are using. All subsequent TM measurements are performed by sending 1440 random inputs obtained by randomly superimposing all 144 fiber modes. This allows us to drastically reduce the size of the TM and the higher-order tensors without losing significant information. The output fiber modes are simply the outputs generated by sending the fiber modes as inputs. These outputs are orthogonal since they correspond to the first 144 output singular vectors of the TM measured in the pixel basis.

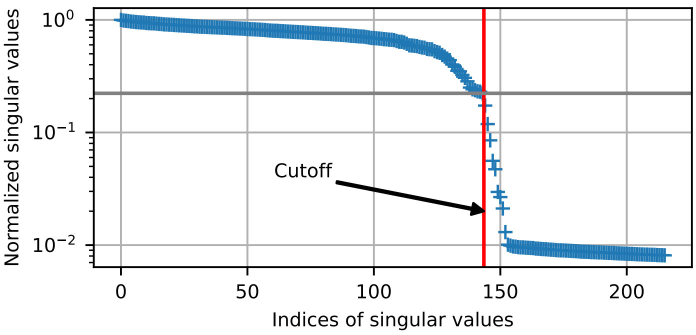

Fig. S2 | Identifying the fiber modes. Singular values of the TM measured in the pixel basis. The fiber modes are identified as the singular vectors for which the singular values light in the plateau. The cutoff value is marked by the vertical red line.

To determine the third-order tensor $\mathcal{W}^{(3)}$, we measure two TMs, $\mathbf{H}^{(\pm)}$, for two different values of the deformation, $\zeta_i \pm d\zeta/2$, centered around the reference value ζ_i. Then, these two measurements can be used to approximate the derivative with respect to ζ using finite differences so that

$$\mathcal{W}^{(3)}_{ijk} = \frac{\mathrm{H}^{(+)*}_{ij}\mathrm{H}^{(+)}_{ik} - \mathrm{H}^{(-)*}_{ij}\mathrm{H}^{(-)}_{ik}}{d\zeta}. \tag{S37}$$

A similar approach is taken to construct the fourth-order tensor $\mathcal{W}^{(4)}$.

5 Experimental estimation of the deformation

5.1 Noise characterization

Before performing the experimental estimation of small deformations, we need to verify the veracity of the assumption of Gaussian noise made in this work for our setup. For this, we performed a set of characterization measurements for two values of the input power and the four fields used to perform the estimation. The first one was performed at the highest input power possible without saturating any pixels of the camera and taking care to send the same amount of energy for each input field. The second one was performed using only 20% of the total power used for the first one. For each set, we sent 500 copies of the four fields and used the measured output intensity distributions to compute the mean and standard deviation value of the intensity over the output pixels as shown in Fig. S3. At higher power we can clearly see some intensity-dependent behavior in the standard deviation which is used to characterize the noise, particularly for the MIIC for which the focal spot is clearly visible. At lower power, however, this dependence on the intensity for the noise is almost completely gone with the σ_n values being fairly uniform.

To confirm the assumption of Gaussian noise, Fig. S4 shows the quantile-quantile plot for the measured intensity deviation from its mean value including every pixel, realization and measured field when compared to a Gaussian distribution for high and low intensity values. This plot compares the cumulative distribution functions obtained from the theoretical Gaussian distribution and the data. If the data follows the model then the points obtained (shown as blue dots) should follow a straight line at 45° (shown as a continuous red line). For the high intensity data, we see a clear deviation for positive values where the points clearly take larger values than those predicted by a Gaussian distribution. This means that the distribution for the data has a positive heavy tail which is consistent with the intensity dependent noise that we see in Extended Data Figure 3. However, this deviation disappears at lower intensity values where all the points follow closely the predicted diagonal line, thus demonstrating that our assumption of Gaussian noise is valid.

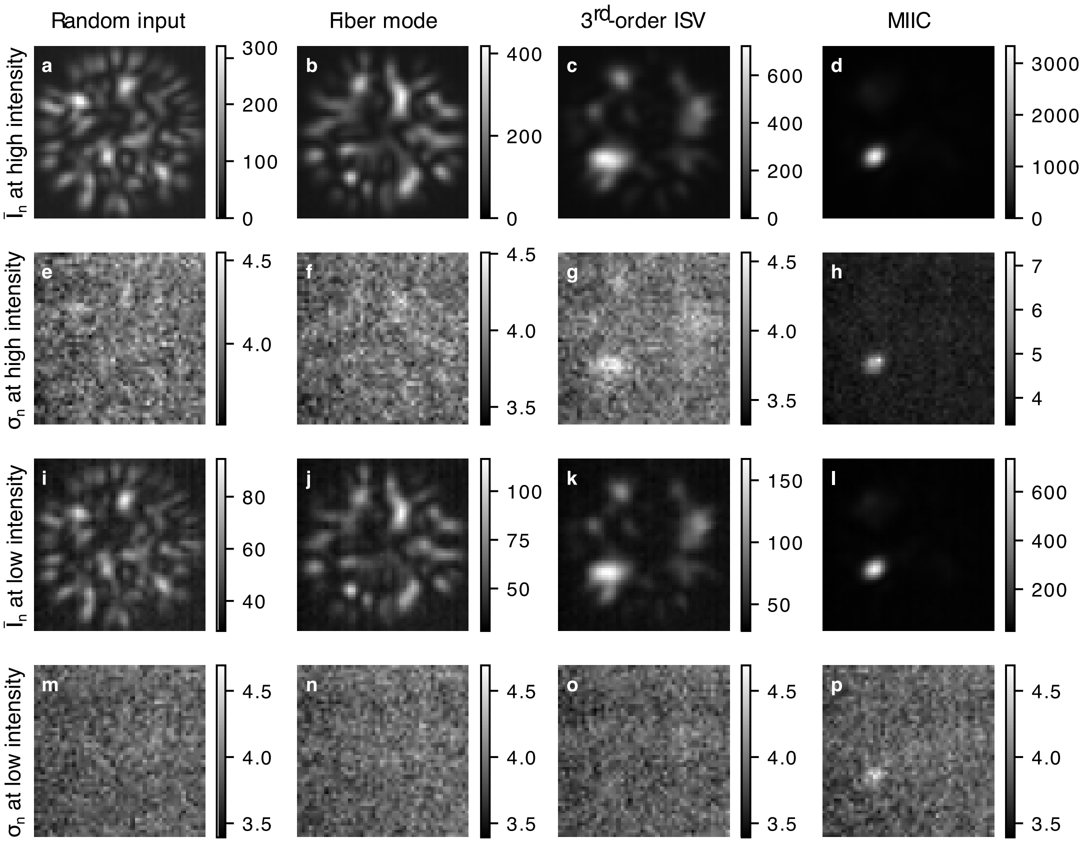

Fig. S3 | Noise characterization. a-d,i-l, Mean intensity distributions and **e-h,m-p,** standard deviations for the four fields used for the parameter estimation at high-intensity values (**a-h**) and when only 20% of the light is sent (**i-p**).

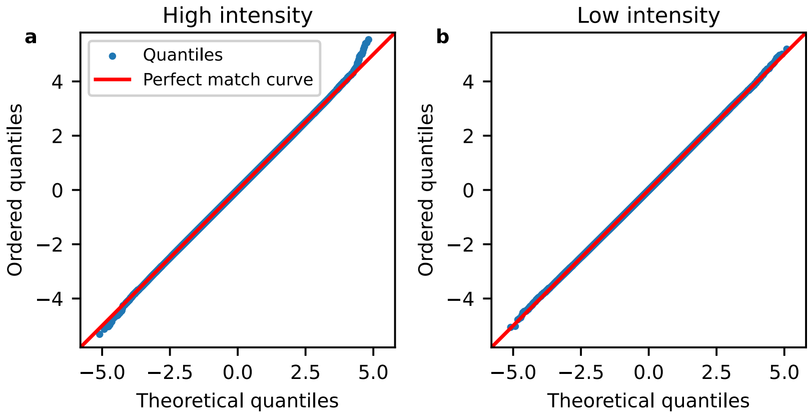

Fig. S4 | Noise QQ plot. **a-b**, Quantile-quantile plot for the measured intensity deviation from its mean value including every pixel, realization and measured field when compared to a Gaussian distribution for high (**a**) and low (**b**) input intensity values.

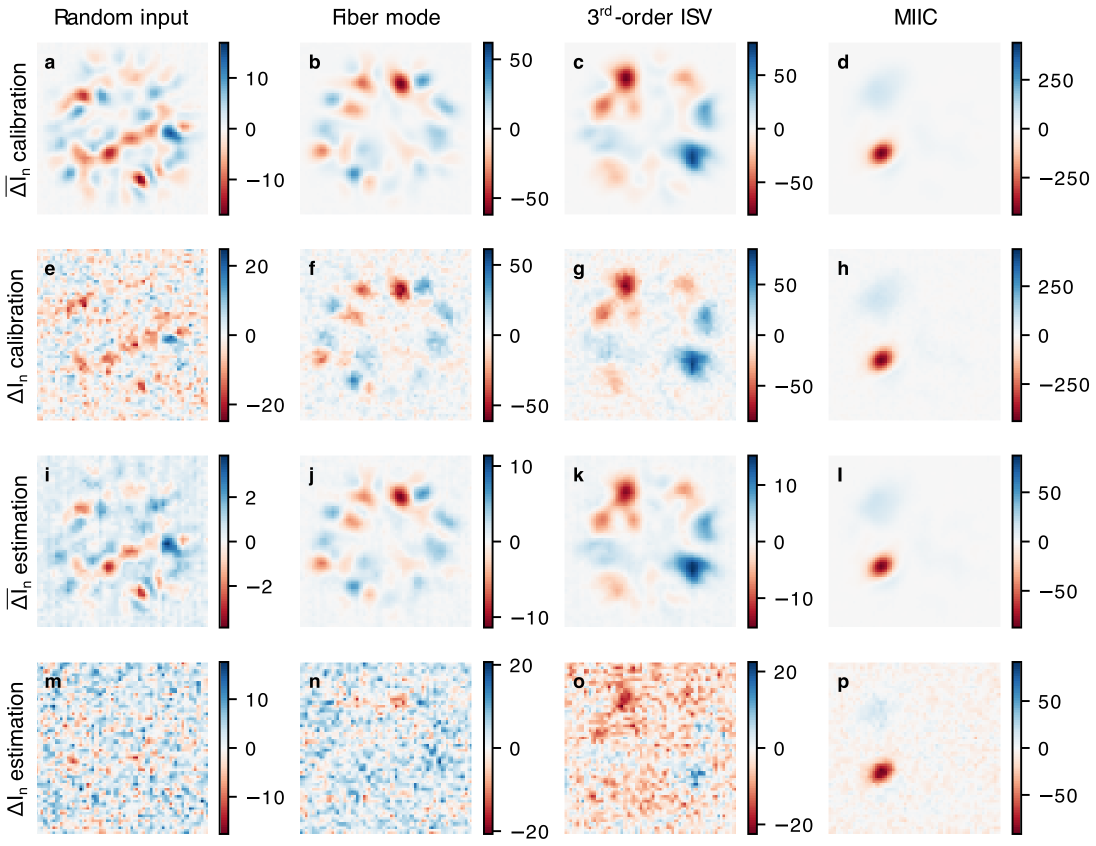

Fig. S5 | Change in intensity. a-d,i-l, Mean and **e-h,m-p,** single change in intensity distributions for the four fields for the measurements taken to calibrate the derivative and acquired at high-intensity values (**a-h**), and the same ones but using only 20% of the light sent for the calibration (**i-p**).

5.2 Estimating the deformation

Given that the perturbations under consideration are small, it is sensible to assume a linear model for the measured intensity distribution χ over the output modes after a change $\Delta\xi$ in the perturbation. Explicitly, we have that

$$\chi \approx \mathbf{I}(\zeta_i) + \partial_\zeta \mathbf{I}(\zeta_i)\Delta\zeta + \boldsymbol{w}(\sigma), \tag{S38}$$

where $\mathbf{I}(\zeta_i)$ represents the output intensity distribution over the output modes prior to changing the deformation, $\mathbf{I}_\zeta(\zeta_i)$ is the derivative of $\mathbf{I}$ with respect to ζ and evaluated at ζ_i, and $\boldsymbol{w}(\sigma)$ is a vector of normally distributed independent random variables with zero mean and standard deviation σ. For this linear model the minimum variance unbiased estimator [1] is given by

$$\Delta\zeta^{(\mathrm{est})}(\chi) = \frac{\partial_\zeta \mathbf{I}(\zeta_i) \cdot [\chi - \mathbf{I}(\zeta_i)]}{\left\|\mathbf{I}_\zeta(\zeta_i)\right\|^2}. \tag{S39}$$

In this model, both the reference intensity distribution $\mathbf{I}$ and the derivative $\partial_\zeta \mathbf{I}$ are assumed to be known. To calibrate the derivative, we first set the input power as high as possible without saturating any pixels for any of the fields used for the estimation. Then, we perform 500 independent measurements for each field and for two deformations centered around the reference value ζ_i. Then, the derivative of

the intensity distribution of each field is estimated by taking the difference between the mean for each deformation and dividing by the total deformation performed between the two points. As can be seen in Fig. S5, the distribution of the mean change in intensity obtained from these calibration measurements is quite smooth, even though the effect of noise is noticeable for each individual realization, and with its main values above the noise level shown in Fig. S3 for all the fields. Also shown in Fig. S5 are the distribution of the mean change in intensity and a single realization at the lower input power used and the same deformations used for the calibration. Here, we can see that each individual change is quite susceptible to noise and that its effect is even noticeable for the mean distribution, particularly for the worst performing field given by the random wavefront. This shows that the calibration at high intensity is only necessary in order to isolate the effects of the inherent sensibility of the input field to the perturbation and those arising from a poor calibration, particularly for the random input for which it is simply not possible to accurately estimate the derivative at a low intensity. For the reference intensity distribution, we can use the same input power used for the estimations since the mean intensity distribution is well above the noise level for all fields (see Fig. S3). Moreover, using the same input power for the reference as for the estimation allows the nonuniform background to be automatically removed.

6 Moving the perturbation along the fiber length

6.1 Fisher information as a function of the location of the deformation

It is interesting to study the dependence of the Fisher information on the specific location of the perturbation. To study this dependence we consider the following model. Given that the deformation is applied at a particular point along the fiber, we can divide our system, and thus the TM, into three part:

$$\mathbf{H} = \mathbf{H}_2 \cdot \mathbf{D} \cdot \mathbf{H}_1, \tag{S40}$$

where $\mathbf{H}_1$ represents the transmission of light up to the region that is deformed, $\mathbf{D}$ represents the propagation through the perturbation, and $\mathbf{H}_2$ the propagation through the final stretch following the deformation. Propagation through small stretches of an unperturbed fiber of length Δz can be modelled by the diagonal matrix, $\mathbf{P}(\Delta z) = \exp(\mathrm{i}\boldsymbol{\beta}\Delta z)$, where $\boldsymbol{\beta}$ is the diagonal matrix containing the propagation constants of the modes of the fiber. Therefore, one way to model the displacement of the deformation is by applying the operator $\mathbf{P}(\Delta z)$ representing the axial shifts to reduce the propagation distance on one side and increase it on the other side

$$\mathbf{H}(\Delta z) = \mathbf{H}_2 \cdot \mathbf{P}(\Delta z) \cdot \mathbf{D} \cdot \mathbf{P}(-\Delta z) \cdot \mathbf{H}_1. \tag{S41}$$

Given that the length of the fiber is fairly small and the deformation not too strong, the matrices $\mathbf{H}_2$ and $\mathbf{H}_1$ are mainly diagonal matrices (see e.g. Refs. [11, 12]). Therefore, it is safe to assume that $\mathbf{H}_2$ and $\mathbf{H}_1$ commute with $\mathbf{P}(z)$ thus giving

$$\mathbf{H}(\Delta z) = \mathbf{P}(\Delta z) \cdot \mathbf{H}_2 \cdot \mathbf{D} \cdot \mathbf{H}_1 \cdot \mathbf{P}(-\Delta z). \tag{S42}$$

With this model we can compute how the Fisher information evolves as we change the location of the perturbation for the various channels that were derived in the main manuscript for the location $\Delta z = 0$. The results are shown in Fig. S6. These plots show that the optimal fields found at Δz remain quite sensitive within a range of 0.5 mm. Depending on the application, this can be more than enough to account for inaccuracies during its implementation. These results are perfectly suited to enhance the precision of point sensors. This type of sensor is designed for a specific parameter and at a specific location, which is what we consider. In this case, a significant drop in the Fisher information as we move away from the region of interest would be beneficial for these sensors since it would make them less sensitive to perturbations happening in other parts of the system.

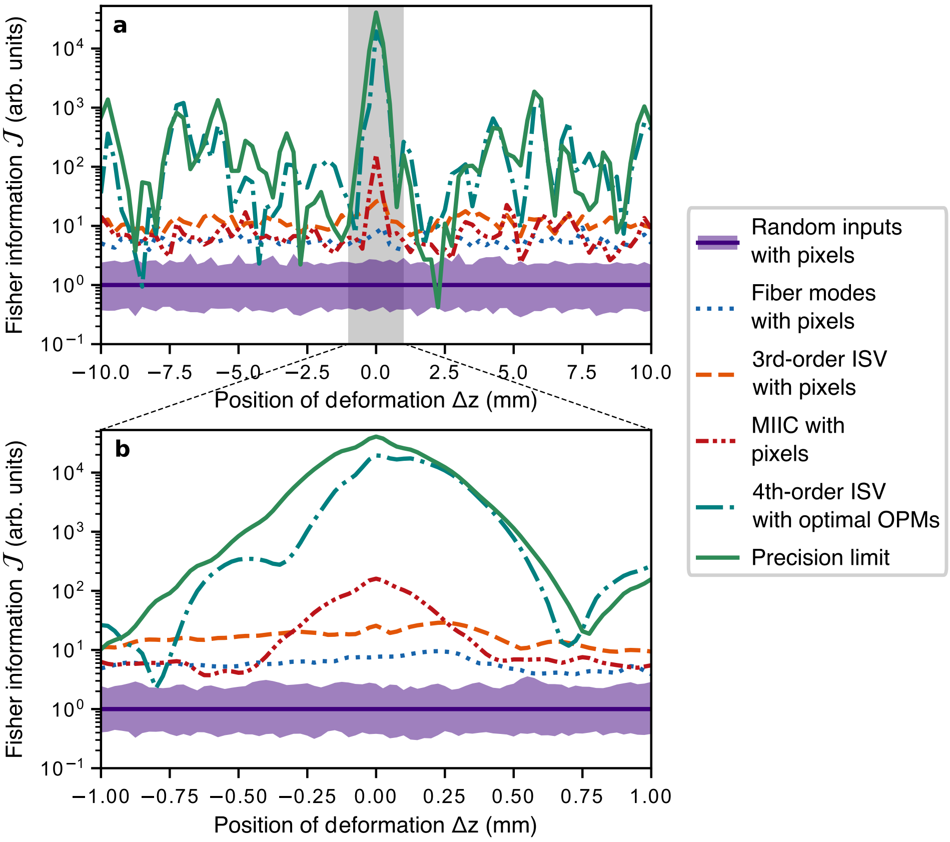

Fig. S6 | Changing the location of the perturbation. **a**-**b**, Evolution of the Fisher information with respect to the location of the perturbation for one-thousand random inputs (continuous line show the mean and the shaded region the full range), the maximum value of the fiber modes, the maximum value of the third-order ISVs, the MIIC, the channel using a single output projection mode, and the channel allowing reaching the precision limit by tailoring both the input and output. Note that for each value Δ the mean value for the random inputs has been used to rescale the others to simplify the comparison. Two different ranges for Δz are shown, a large range of 2 cm (**a**) and a smaller range of 2 mm (**b**).

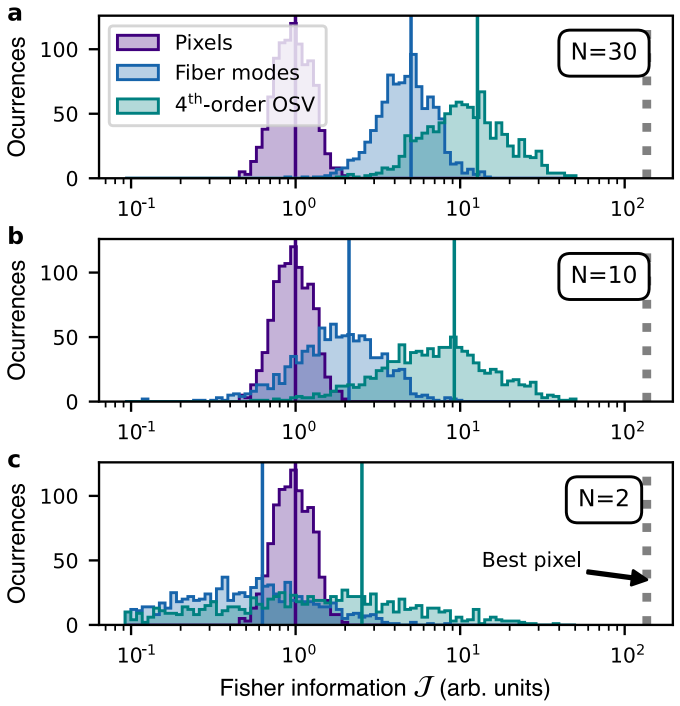

Fig. S7 | Blind estimation. **a-c**, Histogram and mean value (vertical lines) of the Fisher information for 1000 random inputs when projecting the output field onto the pixel basis, the fiber modes, and the output higher-order singular vector. The number of modes used at the output for the fiber modes and the fourth-order OSVs is **a**, 30, **b**, 10, and **c**, 2.

If, instead, the goal is to develop a distributed sensor which can detect perturbations with high sensitivity along the full length of the fiber then the solutions shown in the main manuscript will not be optimal. Nonetheless, our formalism can help address this problem as well. From Fig. S6 it can be seen that the third-order ISV actually provides us with a channel that remains more sensitive than the fiber modes even for large variations of the position of the deformation. This shows that it should be possible to find an input field that is optimal for sensing perturbations irrespective of their location and that the HOSVD provides us again with an excellent starting point, showing again the usefulness of the techniques we are introducing.

6.2 Optimal blind estimation output projection modes

Another thing that can be noted from Fig. S6 is that the channels for which the OPMs have been optimized exhibit larger variations in their Fisher information as a consequence of their specificity to the deformation and specific output field. Much less noticeable variations are present for the channels using the pixel basis as OPMs because this basis is not optimized for any field in particular. These features point to another strategy for dealing with distributed perturbations in which optimal OPMs that do not depend specifically on the input field need to be found. Using the fourth-order output singular vectors (OSVs) of the fourth-order tensor $\mathcal{W}^{(4)}$ as OPMs, it is possible to boost the precision for any input field on average. Figure S7 shows the histograms and mean values obtained when computing the Fisher information for one thousand random inputs when the OPMs are the pixels of a camera, the modes of the fiber, and the fourth-order OSVs.

The benefits of using other OPMs compared to the pixels of the camera become obvious. Even when just 10 of the fiber modes or the fourth-order OSVs are used, they significantly increase the mean value

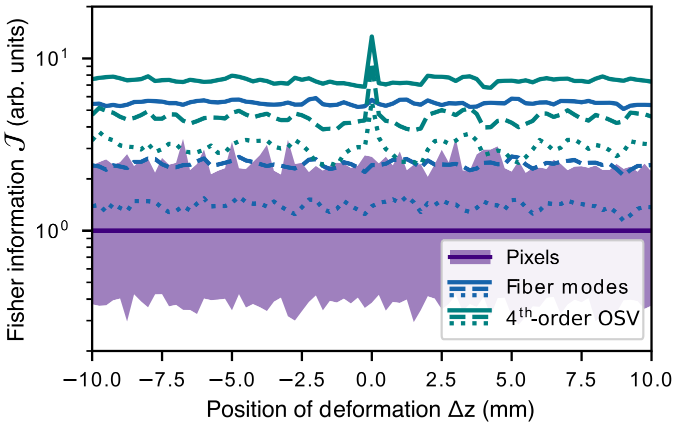

Fig. S8 | Blind estimation with changing location. Mean value of the Fisher information obtained for one-thousand random input fields using as OPMs the pixels of a camera, the fiber modes and the fourth-order OSVs. For the pixel basis the shaded region indicates the range obtained and for the fiber modes and the fourth-order OSVs three different curves are shown for each, the continuous line uses 30 elements, the dashed line uses 10, and the dotted line only 5.

with respect to that obtained with the pixel basis. In particular, the fourth-order OSVs exhibit a much higher mean Fisher information than the one obtained with the fiber modes, and remain a better candidate even when only two of its elements are used as OPMs. Similarly to what we did before, it is also possible to study their behavior as we change the position of the deformation. The results are shown in Fig. S8 where it can be seen that while there is still a clear dependence on the specific location of the deformation, the fourth-order OSVs provide a better alternative to the fiber modes and the pixels of a camera throughout a large displacement range of the perturbation.

References

[1] S. K. Sengupta and S. M. Kay. *Fundamentals of Statistical Signal Processing: Estimation Theory* (JSTOR, 1995).

[2] P. Réfrégier. *Noise Theory and Application to Physics* (Springer New York, 2004).

[3] L. D. Lathauwer, B. D. Moor and J. Vandewalle. On the best rank-1 and rank-$(r_1, r_2, \ldots, r_n)$ approximation of higher-order tensors. *SIAM J. Matrix Anal. Appl.* **21**, pp. 1324–1342 (2000).

[4] L. D. Lathauwer, B. D. Moor and J. Vandewalle. A multilinear singular value decomposition. *SIAM J. Matrix Anal. Appl.* **21**, pp. 1253–1278 (2000).

[5] T. G. Kolda and B. W. Bader. Tensor decompositions and applications. *SIAM Review* **51**, pp. 455–500 (2009).

[6] P. Comon, X. Luciani and A. L. F. de Almeida. Tensor decompositions, alternating least squares and other tales. *J. Chemom.* pp. 393–405.

[7] T. G. Kolda and J. R. Mayo. An adaptive shifted power method for computing generalized tensor eigenpairs. *SIAM J. Matrix Anal. Appl.* **35**, pp. 1563–1581 (2014).

[8] W.-H. Lee. III computer-generated holograms: Techniques and applications. In *Progress in Optics*, pp. 119–232 (Elsevier, 1978).

[9] R. Gutiérrez-Cuevas and S. M. Popoff. Binary holograms for shaping light with digital micromirror devices (2023). E-print.

[10] E. Cuche, P. Marquet and C. Depeursinge. Spatial filtering for zero-order and twin-image elimination in digital off-axis holography. *Appl. Opt.* **39**, pp. 4070–4075 (2000).

[11] M. Plöschner, T. Tyc and T. Čižmár. Seeing through chaos in multimode fibres. *Nat. Photonics* **9** no. 8, pp. 529–535 (2015).
[12] M. W. Matthès et al. Learning and avoiding disorder in multimode fibers. *Phys. Rev. X* **11**, p. 021 060 (2021).
